# Sowing the Seeds of Taste? A Novel Approach to Investigate the Impact of Early Sweet Exposure on Children’s Dietary Taste Patterns from 12 to 36 Mo

**DOI:** 10.1016/j.tjnut.2025.03.017

**Published:** 2025-03-18

**Authors:** Carina Mueller, Monica Mars, Gertrude G Zeinstra, Corine Perenboom, Ciarán G Forde, Gerry Jager

**Affiliations:** 1 Division of Human Nutrition and Health, Wageningen University and Research, Wageningen, The Netherlands; 2 Wageningen Food & Biobased Research, Wageningen University and Research, Wageningen, The Netherlands

**Keywords:** sweet taste, young children, food intake, dietary assessment, complementary feeding

## Abstract

**Background:**

Early food experiences shape children’s eating behavior. Whether initiating complementary feeding (CF) with sweet-tasting foods impacts the taste of later dietary patterns remains unknown. This study combined a quantitative taste intensity database with dietary assessment methods to investigate this.

**Objectives:**

This study aims to investigate whether initiating CF in infants with sweet compared with neutral-tasting foods leads to different dietary taste patterns at 12–36 mo.

**Methods:**

A total of 246 Dutch infants (age 20.2 ± 1.8 wk, 129 girls) participated in an randomized control trial; they received either sweet-tasting (*n* = 125) or neutral-tasting (*n* = 121) fruit and vegetable purees during the first 15 d of initial CF. Dietary intake was assessed at 12, 18, 24, and 36 mo using 3 24-h recalls. Reported foods (*n* = 1277) were grouped into 5 clusters—"sour-sweet," "sweet-fatty," "fatty-salty," "fatty," and "neutral" tasting foods—based on their taste intensity values using K-means clustering. Dietary taste patterns were calculated as the average daily intake of energy (%kcal) and weight (%grams) from each taste cluster and compared between intervention groups.

**Results:**

Overall, children’s energy intake from neutral-tasting foods decreased from 61% ± 11% at 12 mo to 44% ± 12% at 36 mo (*P* < 0.001). Weight intake from neutral foods also declined (from 74% ± 9% to 62% ± 13%, *P* < 0.001). Conversely, children’s energy intake from sweet-fatty, fatty-salty, and fatty foods increased significantly over the study period (from 12% ± 7% to 21% ± 10%, from 8% ± 6% to 13% ± 7%, and from 7% ± 5% to 11% ± 6%, respectively, all *P* ≤ 0.01). No differences were observed between the 2 intervention groups.

**Conclusions:**

Overall, children’s diets became more diverse and intense in taste but exposure to sweet taste during early CF did not influence the dietary taste patterns in later childhood.

**Trial registration number:**

This trial was registered at clinicaltrials.gov as NCT03348176.

## Introduction

Complementary feeding (CF) is an important phase in the development of early-life taste preferences and marks the timing of the initial exposure of infants to a wider variety of foods, after a period of exclusive (breast) milk feeding [[1], [2], [3], [4]]. Foods introduced at initial CF, typically around 4–6 mo, are suggested to influence children’s later food, flavor, and taste preferences [5,6]. Several studies have specifically investigated how the early introduction of fruits and vegetables influences later acceptance or consumption of these foods. A positive correlation was found between the early (between 6 and 12 mo of age) introduction of fruits and later (around 18 mo till preschool age) fruit consumption [[7], [8], [9]], and also between early introduction of vegetables (at the start of CF) and later (up till 2 y of age) vegetable acceptance [9,10]. Several other studies also reported that repeated fruit or vegetable exposure in the first 4–9 mo can significantly increase children’s acceptance and consumption of those foods [[11], [12], [13]]. Hence, the early stages of CF represent a window of opportunity for young children to either learn to accept tastes they innately dislike, such as bitter and sour, or to solidify their preference for tastes they naturally like, such as sweet, or those they adopt early on, such as salty and umami [10,[14], [15], [16], [17], [18], [19]]. However, less is known about how long-lasting these early taste exposures are and whether they can influence later food acceptance and intake [20].

Profiling dietary patterns by the predominant taste properties of the food consumed and the relative contribution of different taste combinations to energy intake offers a new approach to understanding how sensory cues influence habitual dietary patterns [21,22]. Dietary taste patterns are defined as the amount of food coming from foods and beverages with basic tastes (sweet, sour, bitter, salty, and umami) and fat sensations. Dietary taste patterns can provide insights into the predominant taste properties associated with dietary intake patterns, and offer a novel approach to compare differences in energy and food group consumption, based on the tastes people are frequently exposed to [22]. In the first year of life, the majority of children’s daily energy intake comes from foods with low taste intensities—so-called "neutral" tasting foods. In the second year, the diet becomes more varied, with the introduction of complementary foods and the transition to table foods from the family diet. This results in an increase in taste variety and intensity, reaching similar levels to the dietary patterns observed in adults [23,24]. It is, however, unclear whether the variation in terms of taste in children’s diets at 2 y old has been shaped by their early-life taste exposures during the start of CF, and later into the first years of life. Sweet tastes are often used to encourage acceptance of novel complementary foods, but whether this has a lasting impact on subsequent taste preferences and dietary patterns remains unclear. It remains uncertain whether early exposure to sweet tastes affects children’s taste preferences as they grow older [23,25]. This is important given the ongoing debate about the role of repeated exposure to sweetness in shaping children’s taste preferences, liking, and intake of sweet-tasting foods [26,27]. There has been limited research demonstrating that early-life taste exposure has a long-term impact on children’s later food preferences and dietary intake patterns [4], yet it is widely held that exposure to sweet-tasting foods enhances later sweet liking and intake of sugar-containing foods [28].

This study aimed to investigate whether early exposure to sweet-tasting compared with neutral-tasting foods during CF initiation impacts children’s dietary taste patterns at a later age. Specifically, we examined whether children exposed to fruits and sweet vegetables (representing "sweet taste" exposure) or nonsweet vegetables (representing "neutral taste" exposure) for 15 consecutive days during the critical early phase of CF had different dietary taste patterns at 12–36 mo, as measured by the energy and volume of the food products consumed.

## Methods

### Design

We conducted a secondary data analysis on the food intake data from the Baby’s First Bites study (BFB) [29] by linking it to data on taste properties of a range of foods from the (in Dutch) “Smaak, Vet, Textuur (SVT)” study [21,30]. This way we were able to describe children’s dietary taste patterns at 12, 18, 24, and 36 mo.

The BFB protocol was approved by the Ethical Review Board of Education and Child Studies, Leiden University (protocol number ECPW-2015/116) and the Medical Ethical Committee of Wageningen University and Research (METC-WU protocol number NL54422.081.15). It was registered at the Netherlands National Trial Register (identifier NTR6572) and clinicaltrials.gov (NCT03348176) and was conducted according to the guidelines laid down in the Declaration of Helsinki.

The SVT study was approved by the Human Ethics Review Committee of Wageningen University in the Netherlands (ABR number: NL47315.081.13) and Taylor’s University in Malaysia (Ethics reference number: HEC/2015/SBS/023).

### BFB study

The BFB trial examined the effect of vegetable exposure and sensitive feeding practices at the start of CF, on later vegetable acceptance and intake, eating behavior, and weight gain in infants and toddlers. In short, 246 Dutch mother–infant dyads were randomly allocated to one of the following conditions: *1*) repeated exposure to vegetables + no parenting intervention, *2*) repeated exposure to regular sweet-tasting weaning foods + video feedback parenting intervention on sensitive feeding, *3*) repeated exposure to vegetables + video feedback parenting intervention on sensitive feeding, or *4*) repeated exposure to regular sweet-tasting weaning foods + attention-control parenting intervention. The children were allocated to the groups via stratified randomization based on the infant’s sex and their age at the beginning of CF (4, 5, or 6 mo). More information about the BFB study, its design, intervention conditions, and measurements can be found elsewhere [29].

For the current analyses, data from the intervention groups *1*) and *3*), as well as *2*) and *4*) were combined, as the video feedback parenting intervention on sensitive feeding showed no statistically significant effect on the main outcomes of children’s vegetable intake or self-regulation of energy intake [31]. Infants in groups *1*) and *3*) further called the “neutral exposure (NEU) group,” received nonsweet vegetable purees whereas infants in groups *2*) and *4*) further called the “sweet exposure (SWE) group,” received fruit and sweet vegetable purees for 15 d at the start of CF [29].

### Participants

The main inclusion criteria for mothers entering the BFB study with their newborns were as follows: being a first-time mother, having a healthy term infant (37–42 wk of gestation), and planning to start CF at 4–6 mo of the child’s age. A more detailed explanation of the inclusion and exclusion criteria can be found elsewhere [29]. The intervention started when parents initiated CF.

### CF intervention

The NEU and SWE interventions consisted of mothers spoon-feeding their child with either neutral-tasting or sweet-tasting foods for 15 consecutive days at the start of CF at home. In addition to the intervention, infants continued to receive their usual breastmilk or formula feeding. Before the intervention started, all mothers fed their infants neutral-tasting rice flour porridge for a minimum of 5 d to get the child used to spoon-feeding. During the 15-d intervention period, the NEU group received age-appropriate jars of pureed green beans, cauliflower, spinach, and broccoli. The SWE group received age-appropriate jars of pureed apples, pears, bananas, and carrots. Table 1 shows the taste intensity values of the intervention foods derived from the SVT taste panel at the time of the BFB study. After the 15-d intervention, the NEU and SWE group received 100 additional jars of neutral or sweet-tasting age-appropriate purees, as a stimulus to continue the different taste exposures (Supplemental Table 1). These jars could be used at the family’s discretion until the baby was 12 mo old. All provided fruit and vegetable purees were commercially available products (Olvarit, Nutricia).

**TABLE 1:** Mean taste intensity values (0–100) of the intervention fruits and vegetables, obtained from the SVT database [30].

Mean taste intensity (on a scale from 0 to 100)						
Product	Sweet	Sour	Bitter	Umami	Salty	Fatty
Neutral exposure group						
Green beans	7	9	5	8	4	11
Cauliflower	4	5	5	8	3	11
Spinach	7	4	8	3	4	7
Broccoli	5	8	8	9	4	10
Sweet exposure group						
Carrot	29	10	2	6	3	12
Apple	24	26	1	1	3	9
Banana	33	24	1	1	4	14
Pear	24	24	2	1	3	9

Abbreviation: SVT, Smaak, Vet, Textuur.

### Dietary intake

Mothers reported their child’s food intake at 12, 18, 24, and 36 mo through self-administered, web-based 24-h recalls on 3 randomly assigned, nonconsecutive days within a 3-wk period using the online program Compl-eat. The program uses a multiple-pass method and was adapted to infants’ and young children’s diets for the BFB study [32,33]. Subsequently, energy and nutrient intake was calculated by using the Dutch food composition table [34]. More details about the 24-h recalls can be found elsewhere [29].

### Taste database

To assess the children’s dietary taste patterns, average taste intensity values were derived from the SVT project that entails a Dutch and Malaysian database [21,30] of foods rated for their basic taste intensities and perceived fat sensation. To compile this database, a similarly trained adult Dutch and Malaysian sensory panel systematically profiled a series of foods on the intensities of the basic tastes (sweet, sour, bitter, salty, and umami) along with the perception of fat sensation. This evaluation was conducted on a 100-mm visual analog scale using references from sapid solutions and reference foods [21].

For the Dutch dataset, 476 frequently consumed food products were selected and profiled [21]. The foods were selected following their reporting frequency and contribution to the overall energy intake in the Dutch population [22] based on data from the Dutch National Food Consumption Survey of 2007–2010 [34]. We used expert knowledge from research dietitians to select one of the most often consumed brands for profiling. These 476 foods contributed in total to 83% of energy intake for an individual day of consumption in the Dutch population [22]. For the Malaysian dataset, 423 products frequently consumed in Malaysia were profiled; this selection was also based on food consumption data from Malaysia [21]. The Malaysian dataset was used as some products were included in the Malaysian dataset but not in the Dutch dataset, for example, coconut milk. Despite different cultural and geographical locations, the equally trained panels showed similar taste values, as shown by the control foods which were profiled by both panels [21]. A comprehensive explanation of the databases’ development, recruitment, and training regimens for sensory panelists, and the protocols employed for taste profiling are described in detail elsewhere [21].

### Children’s dietary taste patterns

**FIGURE 1:**
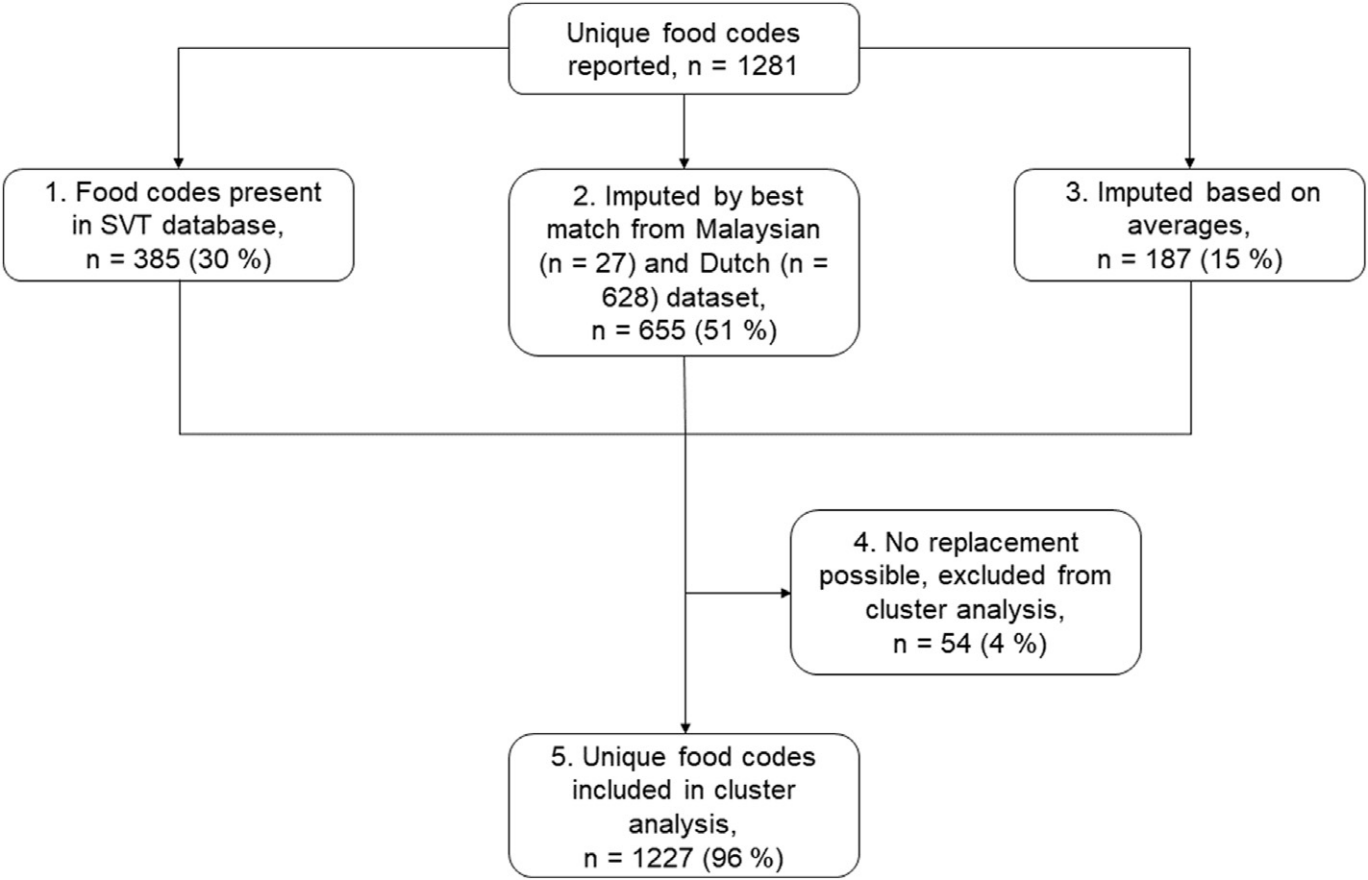
Flowchart of foods reported and steps (1–5) taken to assign taste values to food codes. SVT, Smaak, Vet, Textuur.

In total, 1281 unique food codes were reported in the 24-h recalls (Figure 1). Of these, taste values from 385 foods were derived from the SVT database (step 1). Of the remaining codes that were not rated in the SVT database (*n* = 896), the taste intensity values were imputed by values from matching foods from the SVT database (*n* = 628) or the Malaysian (*n* = 27) database (step 2). For example, if pasta was reported as uncooked, the taste values from boiled pasta were imputed. Further, for 187 foods, taste intensity values were imputed based on the average taste intensity values of similar foods or food categories (step 3). For example, values for canned strawberries were imputed from the average taste intensity values of all canned fruits. Finally, 54 reported foods were excluded from the analyses as there were no appropriate matches for imputation by replacement or imputation (step 4). This group consisted of herbs, spices, and culinary ingredients such as flour that were impossible to profile for the taste database. As a result, a total of 1227 unique food codes (96% of all unique codes) were used for the cluster analysis to form groups of similar-tasting foods (step 5). A list of the reported codes and the imputations can be found in the Supplemental Table 2.

### Taste clusters

Groups with similarly tasting foods, the so-called taste clusters, were based on taste intensity values and fat sensations of all reported products (*n* = 1227) from the 24-h recalls. The taste intensity values were first scaled, and then K-means clustering with Euclidean distance was used to identify distinct subgroups, for example, taste clusters. K-means clustering algorithm builds nonoverlapping clusters by minimizing the total within-cluster variation. This analysis resulted in 5 distinct taste clusters.

On the basis of the most dominant mean taste intensity values for each cluster, we labeled them as “sour-sweet,” “sweet-fatty,” “fatty-salty,” “fatty,” and “neutral.” To ensure consistency across ages, we initially applied this approach separately for each time point, focusing on foods reported exclusively at 12, 18, 24, and 36 mo of age. This approach resulted in similar clusters, which reinforced our decision to build clusters using foods that were reported at all time points. A complete list of the reported foods and cluster assignments can be found in Supplemental Table 2.

### Food intake per taste cluster

The proportional energy intake from each of the 5 taste clusters was calculated at each timepoint. First, the food intake was averaged over the 3 24-h recalls at each time point per child to estimate energy intake. Next, the energy and weight consumed from each taste cluster were calculated per time point and child and expressed as a proportion of total daily food and energy intake (all foods together). Finally, average intake per cluster was calculated per time point and compared between the intervention and control groups.

### Statistical analysis

All statistical analyses were performed in R studio, version 4.3.1 for Windows. Data are expressed as mean and SD (±SD) or frequency [*n* (%)] unless specified otherwise. The threshold set for statistical significance was 5% (*P* < 0.05).

Differences between intervention groups were tested by means chi-square tests in the case of categorical variables, and independent-sample *t* tests in the case of continuous variables. Because of the non-normality, the Welch 2-sample *t*-test was used to assess the differences between 12 and 36 mo in total dietary intake (in energy and weight), energy density of the total diet, and number of unique products consumed. To improve normality, the proportion of energy and weight consumed from the different clusters (dependent variables) was square root transformed. Mixed models for repeated measures (MMRM) [35,36] were used to test whether children’s dietary intake from the taste clusters was different between intervention groups at 12–36 mo. Fixed factors in the model were taste cluster, intervention group, and time point, participant number was included as a random factor, and compound symmetry was used as covariance structure. As mothers’ age differed between the intervention groups, it was added as a covariate to the models; however, it was removed from the final models as it was neither significant nor influenced the outcome values. Fixed effects and their interactions were tested with Analysis of Variance (ANOVA) Type II Wald chi-square test, and pairwise post hoc comparisons were performed with Bonferroni correction.

## Results

### Participants

In total, 246 mother–infant pairs were included (Table 2). The infants in the intervention groups had similar characteristics, without differences in sex distribution, age, BMI-*Z*-score, or the duration of breastfeeding (all *P* >0.05). Mothers in the SWE group tended to be older (Δ 1.2 y; *P* = 0.05) than mothers in the NEU group, but BMI and educational level were not significantly different (*P* > 0.05).

**TABLE 2:** Baseline characteristics of mother–infant pairs, separated by the intervention groups.

	Total (*n* = 246)	NEU group (*n* = 121)	SWE group (*n* = 125)
Infants			
Sex, *n* (%)			
Female	129 (52.4)	65 (53.7)	64 (51.2)
Male	117 (47.6)	56 (46.3)	61 (48.8)
Age^1^ (wk)	20.1 ± 3.9	20.2 ± 1.8	20.0 ± 5.2
BMI-*Z*-scores^2^	−0.23 ± 1.02	−0.23 ± 0.98	−0.24 ± 1.06
Breastfeeding duration, median (range), wk^3^	13 (0–26)	16 (0–26)	12 (0–26)
Mothers			
Age^4^ (y)	30.9 ± 4.9	30.3 ± 4.9	31.5 ± 4.5^5^
BMI^6^ (kg/m^2^)	27.1 ± 5.5	26.8 ± 5.2	27.3 ± 5.8
Education, *n* (%)			
Master’s degree or higher	48 (19.5)	24 (19.8)	24 (19.2)
Bachelor’s degree	95 (38.6)	45 (37.2)	50 (40.0)
Secondary vocational education	78 (31.7)	42 (34.7)	36 (28.8)
Lower than secondary vocational education	25 (10.2)	10 (8.3)	15 (12.0)

Values are shown in means ± SDs or in frequencies (%).

Abbreviations: NEU group, neutral exposure group; SWE group, sweet exposure group.

1 *n* = 243, 3× no date of entering the study, NEU group: *n* = 120; SWE group: *n* = 123.

2 WHO standards; *n* = 244, NEU group: *n* = 120; SWE group: *n* = 124.

3 (Nonexclusive) breastfeeding duration was measured at 18 mo of age; *n* = 237: NEU group: *n* = 117; SWE group: *n* = 120.

4 Significant difference

5 between NEU and SWE group (*P* = 0.05); no difference between groups in any other variable.

6 *n* = 242, NEU group: *n* = 118; SWE group: *n* = 124.

### Changes in dietary taste pattern over time (12–36 mo)

The ANOVA Type II Wald chi-square tests on the MMRM showed a significant main effect of the clusters [χ^2^(4) = 9793.36, *P* < 0.001], time points [χ^2^(3) = 30.12, *P* < 0.001], and the interaction between clusters and time points [χ^2^(12) = 376.38, *P* < 0.001] for the proportional energy intake from the different taste clusters (model 1). Similarly, for the proportional intake in grams (model 2), significant main effects of the clusters [χ^2^(4) = 29 596.86, *P* < 0.001], time points [χ^2^(3) = 44.01, *P* < 0.001], and the interaction between the cluster and time point [χ^2^(12) = 189.71, *P* < 0.001] were observed. No main effect of the intervention or an interaction between intervention, cluster, and time points was found (all *P* > 0.05). This indicates that children’s dietary taste patterns in terms of proportional energy intake and grams intake from the 5 taste clusters changed over time, but this was independent from the child’s exposure to sweet or neutral-tasting foods at the start of CF.

**FIGURE 2:**
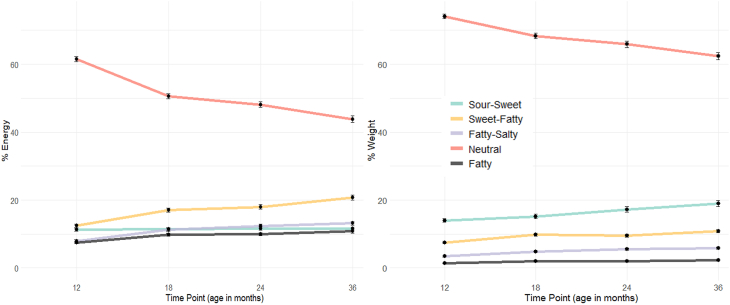
The mean percentage (±SEM) of energy (in kcal) and weight (in grams) consumed from the 5 different taste clusters at 12, 18, 24, and 36 mo of age.

At 12 mo of age, children across both intervention groups consumed 61% ± 11% of their total daily energy and 74% ± 12% of their total daily consumed grams from neutral-tasting foods (Figure 2). Pairwise comparisons showed that children consumed less energy (estimate = −1.24, SE = 0.10, *t* = 12.62, *P* < 0.001) and grams from neutral-tasting foods (estimate = −0.75, SE = 0.09, *t* = 8.44, *P* < 0.001) at 36 compared with 12 mo. However, their energy intake and consumption of grams for sweet-fatty foods increased from 12 to 36 mo (energy: estimate = 1.05, SE = 0.10, *t* = 10.65, *P* < 0.001; grams: estimate = 0.59, SE = 0.09, *t* = 6.62, *P* < 0.001), fatty salty (energy: estimate = 0.80, SE = 0.10, *t* = 7.96, *P* < 0.00; grams: estimate = 0.56, SE = 0.09, *t* = 6.22, *P* < 0.001), as well as for fatty foods (energy: estimate = 0.54, SE = 0.10, *t* = 5.40, *P* < 0.001; grams: estimate = 0.33, SE = 0.09, *t* = 3.65, *P* < 0.01). Furthermore, although their consumption in grams from sour-sweet foods increased over time (estimate = 0.56, SE = 0.09, *t* = 6.28, *P* < 0.001), energy intake from those foods remained stable. Taken together, these results indicate that children’s diets became more varied and intense in taste from 12 to 36 mo of age.

### Children’s energy consumption, intake in grams, and the variability of consumed products at 12–36 mo

Table 3 shows the average daily intake and the number of different products consumed for the 4 timepoints. Paired samples *t* tests showed that children’s average daily energy intake (in kcal) increased by 26% ± 8% (*P* < 0.001) whereas their daily consumed weight (in grams) increased by 7% ± 2% (*P* < 0.001) from 12 to 36 mo. In addition, the diets of children from 12 to 36 mo became more energy dense (*P* < 0.001) and consisted of a greater product variety, following that the number of different products children consumed per day increased from 9 ± 3 to 12 ± 4 products (*P* < 0.001). For all 4 time points, there were no differences between the SWE and NEU groups in any of these outcomes (all *P* > 0.05). Hence, children’s energy intake increased more than the amount (weight) of foods consumed, which is in line with the increase in energy density of the diets.

**TABLE 3:** Average daily intake and number of different products consumed at each timepoint (mean ± SD).

Age (mo)	Energy (kcal)	Volume (g)	Energy density of foods (energy/volume)	Number of unique products consumed (range)
12	932 ± 187	1224 ± 247	0.77 ± 0.13	9 ± 3 (2–19)
18	1051 ± 198	1289 ± 255	0.83 ± 0.13	11 ± 3 (5–25)
24	1076 ± 248	1297 ± 297	0.84 ± 0.16	11 ± 3 (4–19)
36	1170 ± 260	1316 ± 314	0.91 ± 0.18	12 ± 4 (2–28)

## Discussion

We assessed whether exposure to sweet compared with neutral-tasting foods during the initiation of CF affected children’s dietary taste patterns from 12 to 36 mo using a novel approach to compare taste patterns in food intake, by combining dietary assessment with taste intensity data. No effect of sweet compared with neutral taste exposure during CF initiation was found in children’s dietary taste patterns at 12, 18, 24, or 36 mo. However, our results showed that children’s dietary taste patterns evolved over time across the entire study population. Diets became progressively more intense in various tastes, incorporating a broader range of food products. Additionally, the energy density of the diets increased.

Whether repeated exposure to sweet taste can affect later sweetness preferences, liking, and subsequent intake of sweet-tasting foods is an ongoing debate. To date, no research has systematically proven that early-life sweet taste exposure results in higher preference for, and greater intake of sweet foods [27,37,38]. Although some studies reported that repeated exposure to sweet foods in infancy was related to later preference or liking of those specific foods [14,39], it was unrelated to preference or liking of other sweet products [37,39]. Our findings align with these studies and offer evidence supporting the claim that exposure to sweet tastes in early life does not influence later sweet food preferences or intake in children.

Humans are born with an innate preference for sweetness [40], which continues through early childhood and may result in a ceiling effect, making it difficult to increase liking for already liked tastes. This explains why exposure to neutral compared with sweet foods at the start CF did not affect children’s dietary taste patterns, whereas other studies showed that repeated exposure to unfamiliar or initially disliked flavors increased in food acceptance, intake, or preference [41,42]. Early exposure to different infant formulas with distinct sour or bitter tastes was linked to later preferences for similar-tasting apple juices when children were 4–5 y old [41]. This effect was also seen after the milk feeding phase, with 2-y-old children consuming more of initially unfamiliar vegetables after repeated exposure, especially those with innately disliked taste [42]. Thus, early repeated exposure to tastes may only influence dietary patterns for tastes that are inherently disliked, such as bitter or sour-tasting foods.

Although also other recent reviews suggest that sweet taste exposure is unlikely to influence sweetness preference, liking, or subsequent food intake [28,38], it is possible that the intervention did not adequately increase sweet taste exposure in the current study. The 15-d exposure to sweet tastes may have been insufficient in duration, intensity, or differentiation between groups. For the intervention, commercially available age-appropriate fruit and vegetable purees were used to produce 2 taste exposure groups. These purees are designed to be palatable for infants, which may result in a moderate taste profile. However, if only foods that are significantly sweeter or more bitter than those regularly consumed by infants could affect later dietary taste patterns, the ecological validity of such an intervention would be questionable.

We found that children’s diets became progressively more intense and diverse in taste qualities, with a greater variety of food products consumed, and those products becoming more energy dense. The proportion of energy and grams from neutral-tasting foods decreased, whereas the proportions from sweet-fatty, fatty, and salty-fatty foods increased. This aligns with previous research findings. Two studies in 1–2 y old and 2–3 y old Dutch children showed that children’s dietary taste patterns around the age of 2 become more similar to those of Dutch adults [22,23]. The similarity between child and adults’ dietary taste patterns suggests that food preferences formed in early childhood persist into later adulthood [43]. These similarities may also indicate that by the age of 2 y old, children grow into accepting and eating table foods shared by the entire family. This is supported by data from the Feeding Infants and Toddlers Study, showing that after 24 mo, the diet stabilizes and includes less energy from milk and vegetables and more energy from mixed dishes, grains, and sweets, and begins to resemble adult diets [24,44]. In addition, in a study of 3-y olds, it was observed that the liking of different fruits was strongly predicted by whether children knew the fruits from their home setting [45]. Hence, the family home food environment and daily eating habits are likely to have a stronger influence on a child’s food intake patterns and preferences, than early exposure during CF to sweet-tasting foods.

Although the proportion of energy intake from sour-sweet foods remained relatively stable from 12 to 36 mo, children consumed more of these foods in terms of weight over time in our study. This suggests that children’s acceptance of sour tastes increases as they grow older. However, these foods do not make a large contribution to the overall energy intake due to the low energy density of such foods (for example, plain yogurts or sour fruits). Previous studies have not consistently measured the quantity of foods consumed, making it difficult to compare results. Our findings highlight the importance of including the weight of food consumed to obtain a more comprehensive picture of the energy density of the entire diet and to ensure that foods with a low energy density are not neglected.

Our results are based on unique, extensive databases containing taste intensity ratings of food products and longitudinal dietary intake data from young children. Linking these diet and taste databases allowed us to track children’s food acceptance and food intake taste patterns over a critical period during the development of their habitual dietary behaviors. Previous research has depicted dietary taste patterns in adults [22], taste pattern developments in children from 1 to 2 y [23], or the effects of weaning with fruits or vegetables on subsequent acceptance and intake of these foods in childhood [[9], [10], [11], [12], [13],[46], [47], [48]]. However, sweet taste exposure as compared with neutral taste exposure during CF initiation has not yet been linked with children’s later dietary taste patterns. Using a data-driven clustering approach, we were able to analyze longitudinal dietary patterns without prespecifying the taste clusters. We tested several different approaches to clustering based on centroids or cut-offs to ensure robust and optimal taste clusters. Although interesting, caution should be exercised in extending these conclusions further as our study is not without some limitations. The SVT database was created based on perceived taste intensities of rated products from adults, rather than children. Whether young children perceive the taste quality and intensity of food products in the same way is difficult to judge but may contribute some variability to the associations between product taste and dietary patterns. It is also noteworthy that the SVT database did not contain all products consumed by the children in our study, so we relied on “best-match” replacements and imputations of taste intensity values to estimate some foods taste intensities. This may have contributed to additional variability in the relationship between taste ratings and dietary taste clustering. However, children’s energy intake from the different taste clusters was similar to previous research in 1–2 y olds [23]. This supports our approach and shows that different food intake data lead to similar overall taste clusters in a similar age group.

Future studies should replicate our findings and examine the effects of early sweet taste exposure on food preferences and intake beyond the third year of life. Investigating the effect of early-life taste exposure across all basic tastes and over a longer exposure period would provide a more comprehensive understanding of how early influences shape the development of our food preferences and habitual later dietary patterns. Finally, tracking the taste patterns of parents and siblings can offer new insights into the development of a child’s dietary taste pattern and help shed new light on whether children’s intake simply mimics that of their wider family meals and diet when looking from a taste perspective.

In conclusion, we found no effect of initiating CF with sweet or neutral-tasting foods on children’s dietary taste patterns from 1 to 3 y, and conclude there is no effect of introducing sweet foods on subsequent sweetness preference or a higher sweet food intake in later childhood. We observed that children’s diets became more diverse and intense in taste as they increase the variety and energy density of the foods consumed in their diets to support growth in later childhood.

## Author contributions

The authors’ responsibilities were as follows – CM: protocol design, methodology, study conduct, formulating research questions, data analysis, interpretation of findings, writing – original draft, visualization; MM: data analysis plan, writing - review and editing, supervision, methodology; GGZ: writing - review and editing, supervision, methodology; CGF: writing - review and editing, supervision; CP: methodology, data collection, writing - review and editing; GJ: writing - review and editing, supervision, methodology; and all authors: read and approved the manuscript.

## Data availability

The dataset is available on request.

## Funding

The Baby’s First Bites project was funded by the Dutch Research Council (NWO; grant number 057-14-002). The SVT project was funded through Taylor’s University under the Fundamental Research Grant Scheme (Research Grant no. FRGS/1/2013/SS03/TAYLOR/03/1) and supported by Wageningen University and Taylor’s University through PhD fellowships. The current work has been supported by the European Union’s Horizon 2020 research and innovation program under the Edulia project, a Marie Sklodowska-Curie Innovative Training Network program (grant agreement No. 764985) and the TKI Agri & Food, PPS allocation AF17107.

## Conflict of interest

The authors report no conflicts of interest.
