# Supplementary material for: Sowing the Seeds of Taste? A Novel Approach to Investigate the Impact of Early Sweet Exposure on Children’s Dietary Taste Patterns from 12 to 36 Mo
Source: J Nutr. 2025 Mar 18;155(5):1466–73. doi: 10.1016/j.tjnut.2025.03.017 (PMC12121417; doi:10.1016/j.tjnut.2025.03.017)
Supplement: multimedia component 1 [file mmc1.docx]

**Supplementary Table 1.** Infant purees families received after the intervention, four different purees each at 3 different ages between 4 and 12 months. NEU = neutral exposure, SWE = sweet exposure

|  | **4-6 months** | **6-8 months** | **8-12 months** |
| --- | --- | --- | --- |
| **NEU group** | Broccoli  Cauliflower  Green beans  Spinach | Broccoli-turkey-rice  Cauliflower  Green beans-ham-rice  Spinach-fish-rice | Broccoli-chicken-potato  Cauliflower casserole  Green beans-beef-potato  Spinach-beef-potato |
| **SWE group** | Apple  Banana  Carrot  Pear | Apple-banana-orange  Apple-pear  Carrot-chicken-rice  Peach-banana-kiwi | Apricot-apple-banana  Apple-banana-pear  Apple-strawberry-banana  Carrot-chicken-potato |
